# Long Chain Omega-3 Polyunsaturated Fatty Acid Supplementation Protects Against Adriamycin and Cyclophosphamide Chemotherapy-Induced Bone Marrow Damage in Female Rats

**DOI:** 10.3390/ijms19020484

**Published:** 2018-02-06

**Authors:** Chia-Ming Fan, Yu-Wen Su, Peter R. Howe, Cory J. Xian

**Affiliations:** 1 School of Pharmacy and Medical Sciences, and UniSA Cancer Research Institute, University of South Australia, Adelaide, SA 5001, Australia; chia-ming.fan@unisa.edu.au (C.-M.F.); yu-wen.su@unisa.edu.au (Y.-W.S.); 2 Clinical Nutrition Research Centre, School of Biomedical Sciences and Pharmacy, University of Newcastle, Callaghan, NSW 2308, Australia; peter.howe@newcastle.edu.au; 3 Institute for Resilient Regions, University of Southern Queensland, Springfield, QLD 4300, Australia

**Keywords:** breast cancer chemotherapy, anthracycline chemotherapy, bone marrow toxicity, marrow cellularity, marrow adiposity, bone damage, bone marrow damage, long chain omega-3 polyunsaturated fatty acids

## Abstract

Although bone marrow and bone toxicities have been reported in breast cancer survivors, preventative strategies are yet to be developed. Clinical studies suggest consumption of long chain omega-3 polyunsaturated fatty acids (LCn3PUFA) can attenuate age-related bone loss, and recent animal studies also revealed benefits of LCn3PUFA in alleviating bone marrow and bone toxicities associated with methotrexate chemotherapy. Using a female rat model for one of the most commonly used anthracycline-containing breast cancer chemotherapy regimens (adriamycin + cyclophosphamide) (AC) chemotherapy, this study investigated potential effects of daily LCn3PUFA consumption in preserving bone marrow and bone microenvironment during chemotherapy. AC treatment for four cycles significantly reduced bone marrow cellularity and increased marrow adipocyte contents. It increased trabecular bone separation but no obvious changes in bone volume or bone cell densities. LCn3PUFA supplementation (375 mg/100 g/day) attenuated AC-induced bone marrow cell depletion and marrow adiposity. It also partially attenuated AC-induced increases in trabecular bone separation and the cell sizes and nuclear numbers of osteoclasts formed ex vivo from bone marrow cells isolated from AC-treated rats. This study suggests that LCn3PUFA supplementation may have beneficial effects in preventing bone marrow damage and partially protecting the bone during AC cancer chemotherapy.

## 1. Introduction

Breast cancer is known as the most frequently diagnosed cancer in women. In the past decade, treatment for breast cancer has been transformed through changes in biological understanding and clinical presentations of the disease. Apart from surgical treatment, hormonal therapy and radiotherapy, the majority of patients often still require chemotherapy due to the advanced disease. There is no doubt that earlier diagnosis with the correct use of adjuvant treatments have contributed to decreased mortality of breast cancer survivors.

Breast cancer chemotherapy, with combination use of several different drugs, was shown to be more effective than the use of single chemotherapy alone. One of the most commonly used anthracycline-containing chemotherapy regimens is adriamycin (doxorubicin) + cyclophosphamide (AC). Although AC chemotherapy is often omitted for women with low risk node-negative breast cancer, it is still an integral part of the adjuvant treatment for treating node-positive breast cancer. However, despite the efficacy of AC in the treatment of breast cancer, cumulative dose during treatment is known to cause toxicities such as bone marrow damage, cardio-toxicity or congestive cardiomyopathy and potentially bone damage. Bone marrow toxicity or myelosuppression has been commonly reported as the immediate and dose-limiting toxicity in breast cancer survivors. Increased bone marrow toxicity is consistently higher with use of alkylating agents or anthracycline-based regimens in a population-based setting [1]. In addition, bone marrow microenvironment was also found to be severely damaged following several cycles of AC or CEF (cyclophosphamide, epirubicin and 5-fluouracil) chemotherapy in rat models, including a severe reduction in bone marrow cellularity and an increase in marrow adiposity [2,3]. Apart from bone marrow damage, cardio-toxicity as an adverse effect has also been commonly reported [4]. However, there have been very few studies addressing whether AC combination can cause bone damage as a chronic toxicity, although it was reported that pre-menopausal women who developed ovarian failure following chemotherapy are at a risk of rapid bone loss [5]. In addition, recent laboratory studies using female rat models also revealed several cycles of combination chemotherapy with AC or CEF were able to induce small reductions of trabecular bone volume within the metaphysis bone [2,3].

Since increased risk of bone marrow toxicity has been found to be consistently higher in those receiving alkylating agents or anthracycline-based regimens [1], and anthracycline-based combination chemotherapy continues to be the integral part of breast cancer chemotherapy due to their treatment efficacy, it is important to not only understand the underlying mechanisms for anthracycline chemotherapy induced-bone marrow/bone defects, but also to develop potential strategies for protecting bone marrow and bone during breast cancer chemotherapy. Long chain omega-3 polyunsaturated fatty acids (LCn3PUFA), including eicosapentaenoic acid (EPA) and docosahexaenoic acid (DHA), are known to have various health benefits including prevention of cardiovascular diseases and promoting bone health [6]. The health benefits of LCn3PUFA are mainly due to their anti-inflammatory actions. Clinically, a promising association between LCn3PUFA intake and improved bone mineral density has been reported in some studies [7]. Consumption of LCn3PUFA has also been shown to attenuate age-related bone loss [8,9]. Furthermore, animal studies reported that dietary LCn3PUFA supplementation can enhance bone mass and bone formation in ovariectomized rats [10], as well as protecting bone and bone marrow during short-term methotrexate (MTX) chemotherapy in young rats [11,12]. However, it is not known whether LCn3PUFA supplementation can also protect bone and bone marrow during AC chemotherapy.

Due to some similarities in bone/bone marrow outcomes and in some cellular processes (including increased osteoclastogenesis, oxidative stress and bone marrow adipogenesis and decreased osteogenesis) between postmenopausal osteoporosis and cancer chemotherapy-induced bone/marrow damages [11,13,14,15], we hypothesized that LCn3PUFA supplementation, which has shown benefits in protecting bone and bone marrow as reported in the above recent clinical and animal studies, may also have some efficacy in alleviating bone/bone marrow damage caused by combination breast cancer chemotherapy. Using a rat model, the current study addressed whether daily LCn3PUFA supplementation could help to alleviate bone marrow and bone damage during AC combination chemotherapy.

## 2. Results

### 2.1. Treatment Effects on Body Weight Changes

**Figure 1:**
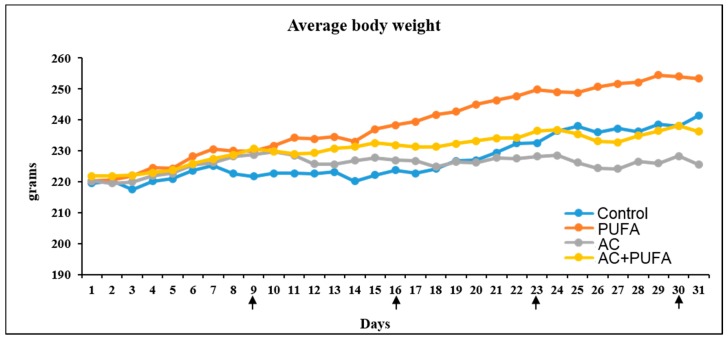
Effects of daily omega-3 polyunsaturated fatty acid (PUFA) supplementation and 4 weekly AC treatment on daily body weights. Vertical arrows indicate days of injections with water control or adriamycin and cyclophosphamide in combination (AC).

Overall, rats from all treatment groups appeared to be healthy and gained weight constantly throughout the trial, although the AC alone group failed to gain weight at a rate of the control rats following the third of 4 cycles of AC treatment (Figure 1) (*p* > 0.05). Interestingly, when comparing both water gavage and LCn3PUFA gavage control groups, rats gavaged with LCn3PUFA appeared to gain more weight when compared to saline control. Similarly, in both groups receiving AC treatment, starting from day 12, AC + LCn3PUFA-treated rats appeared to have a slightly higher weight gain when compared to AC-alone treated rats (*p* > 0.05).

### 2.2. Treatment Effects on Bone Marrow Microenvironment

**Figure 2:**
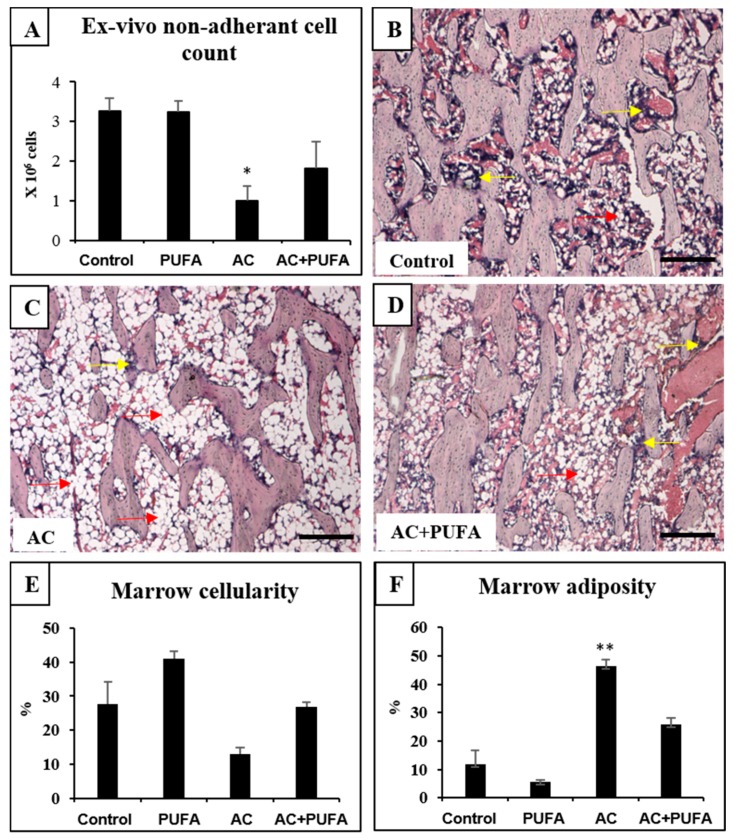
Effects of daily omega-3 polyunsaturated fatty acid (PUFA) supplementation during weekly AC treatment on bone marrow constitution. Treatment effects on counts of bone marrow non-adherent cells isolated from treated rats (**A**); Haematoxylin and eosin (H&E)-stained sections of the lower secondary spongiosa region of the tibia from a control rat (**B**); an AC-treated rat (**C**) and an AC + PUFA-treated rat (**D**); with yellow arrows pointing to non-fat cell bone marrow cells, red arrows pointing to adipocytes. Scale bar on all panels = 200 µm. Treatment effects on the marrow cellularity (%) (**E**) and marrow adiposity % (**F**). Significant values are indicated as: * *p* < 0.05 and ** *p* < 0.01 compared to control.

As a means to evaluate treatment-induced bone marrow damage or potential protection, this study also examined treatment effects on overall bone marrow microenvironment and cellularity. Our ex vivo counting of non-adherent bone marrow cells isolated from the AC-treated rats revealed a significant drop in bone marrow cells when compared to the control (*p* < 0.05) (Figure 2A). Consistently, from histological analyses of haematoxylin and eosin (H&E)-stained tibia sections, this study also observed an obvious trend of depletion of bone marrow cells following 4 cycles of AC treatment within the lower secondary spongiosa when compared to normal control rats (*p* > 0.05) (Figure 2B–E). The depletion of bone marrow cells was accompanied by a significant increase in marrow adipocyte contents (*p* < 0.01) (Figure 2B–D,F).

In rats receiving daily LCn3PUFA supplementation during AC treatment, bone marrow cellularity had a trend of improvement when compared to AC treatment alone (*p* > 0.05) as revealed by ex vivo cell counting (Figure 2A) and by histological analyses (*p* > 0.05) (Figure 2E). Furthermore, daily LCn3PUFA supplementation during AC treatment suppressed AC-induced increase in marrow adipose content to a level that was comparable to that of control rats (*p* > 0.05) (Figure 2F).

### 2.3. Treatment Effects on Overall Trabecular Bone Volume and Structures in the Metaphysis

**Figure 3:**
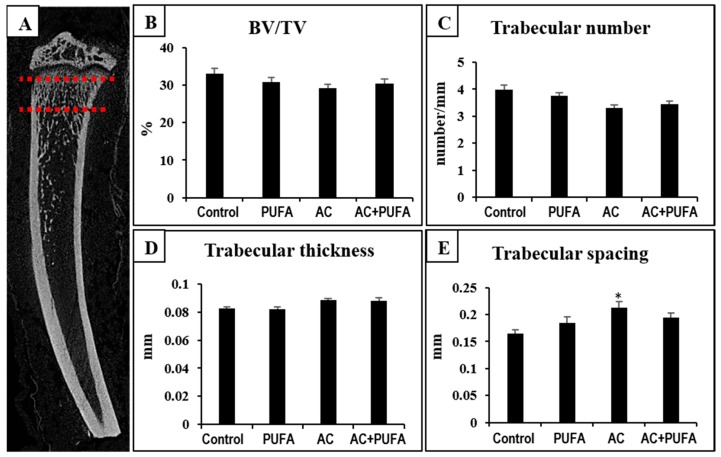
Effects of daily omega-3 polyunsaturated fatty acid (3PUFA) supplementation during weekly AC treatment on metaphyseal trabecular bone volume and structure. μ-CT longitudinal cross-section of a control rat tibia (**A**); Region within the red dotted line (region of interest) represents the first 2 mm of metaphysis from the end of growth plate, where all μ-CT scan data were analysed. Analyses of the treatment effects on trabecular bone volume/tissue volume (BV/TV) (**B**) and trabecular structures including trabecular number (**C**); trabecular thickness (**D**) and trabecular spacing (**E**).

To examine treatment effects on overall trabecular bone volume and structural changes, micro-computed tomography (µ-CT) 3D analyses were conducted on the region 0–2 mm below the growth plate, consisting of mainly primary and secondary spongiosa (regions rich in bone trabeculae) (Figure 3A). Although µ-CT analyses revealed no significant changes in trabecular bone volume across all treatment groups (*p* > 0.05) (Figure 3A,B), some trabecular structural changes were observed. A trend of reduction in trabecular number (*p* > 0.05) and a significant increase in trabecular spacing were observed in AC alone-treated group when compared to the control group (*p* < 0.05) (Figure 3C–E). LCn3PUFA supplementation did not affect AC treatment-induced trabecular structural changes (*p* > 0.05) (Figure 3C–E).

### 2.4. Treatment Effects on Bone Cells at the Metaphysis

**Figure 4:**
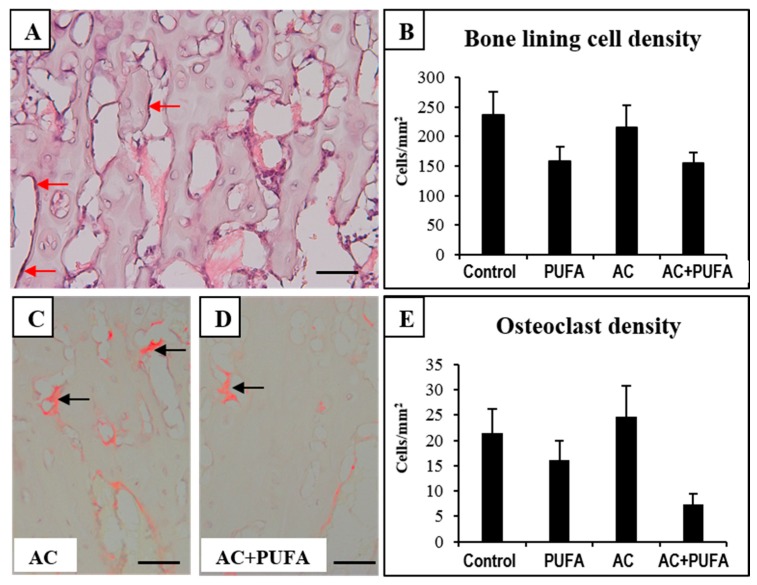
Effects of daily omega-3 polyunsaturated fatty acid (3PUFA) supplementation during weekly AC treatment on bone cells at primary spongiosa. H&E stained metaphysis of a control rat (**A**); with arrows pointing to bone lining cells. Analyses of the treatment effects on bone lining cell density (**B**); Tartrate-resistant acid phosphatase (TRAP)-stained tibia sections of an AC-treated rat (**C**) and AC + PUFA-treated rat (**D**); with arrows pointing to multinucleated osteoclasts. Treatment effects on osteoclast density (**E**). Scale bar on all panels A, C and D = 50 µm.

To analyse the potential cellular basis that may contribute to the bone changes observed following treatments, bone cell densities were examined. Firstly, since BLCs are more abundant than the bone forming osteoblasts in skeletally mature rats, this study examined the treatment effects on densities of bone lining cells (BLCs). BLCs, that appear flattened and extended over trabecular surface, are the inactive forms of osteoblasts and are responsible for protection of the bone surface (Figure 4A), and can be induced to proliferate and differentiate into osteogenic cells [16]. Histological analyses of H&E-stained slides revealed no significant changes of BLC densities across all treatment groups, suggesting treatments had no effect on bone surface protection against external factors which may induce bone damage (Figure 4B).

Apart from osteogenic cells, this study also examined treatment effects on bone resorptive cells osteoclasts. Counting of tartrate-resistant acid phosphatase-positive (TRAP^+^) multinucleated osteoclasts on the trabecular surface revealed no significant overall changes in the osteoclast density between all treatment groups. However, daily LCn3PUFA supplementation during AC treatment showed a statistically non-significant trend of being able to lower the osteoclast density when compared to AC alone-treated rats (*p* > 0.05) (Figure 4C–E).

### 2.5. Treatment Effects on Ex Vivo Osteoclast Formation Potential

**Figure 5:**
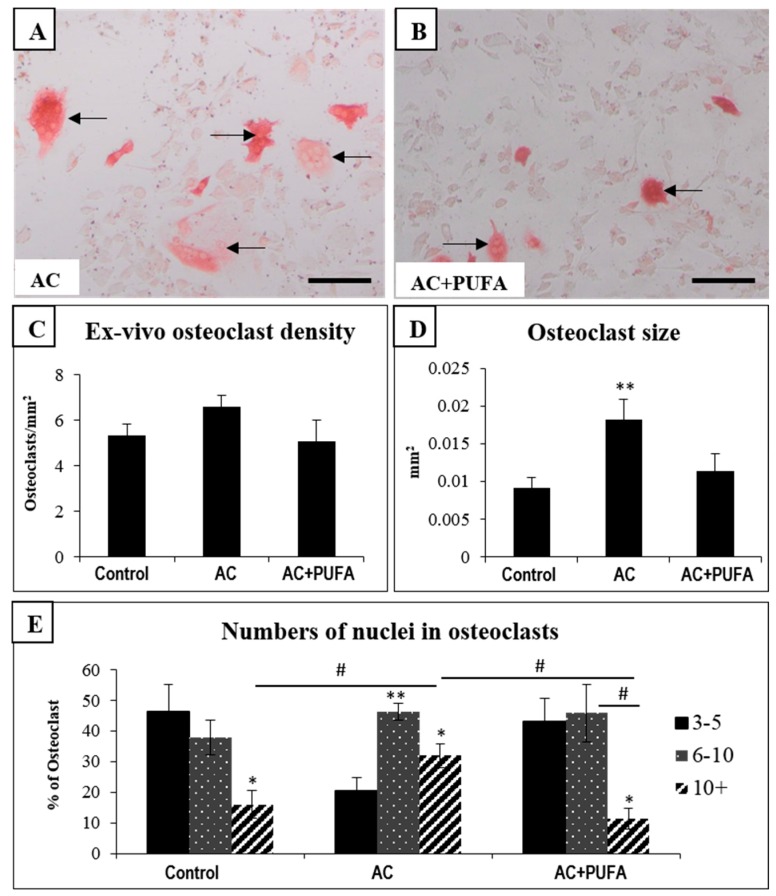
Effects of daily omega-3 polyunsaturated fatty acid (PUFA) supplementation during weekly AC treatment on ex vivo osteoclast formation potential. Ex vivo osteoclast formation from bone marrow cells of an AC-treated rat (**A**) and from bone marrow cells of AC + PUFA-treated rat (**B**); with arrows pointing to multinucleated tartrate-resistant acid phosphatase-stained osteoclasts. Treatment effects on the ex vivo osteoclast formation ability (**C**); size of osteoclasts formed (**D**); and numbers of nuclei within osteoclasts formed (**E**) (* or ** compared to the respective group with 3–5 nuclei). Scale bar on all panels = 100 µm. # above horizontal bars reflects differences between connected groups. Significant values are indicated as: * or # = *p* < 0.05 and ** = *p* < 0.01.

In order to examine whether treatments can have an effect on osteoclast formation potential from the bone marrow cells, an ex vivo formation assay was performed using bone marrow cells obtained from treated rats. In consistency to our histological findings, the ex vivo osteoclast formation assay also revealed no changes in osteoclast formation potential between all treatment groups (Figure 5A–C). Interestingly, however, the sizes of osteoclasts formed from bone marrow of AC-treated rats were significantly larger when compared to the control rats (*p* < 0.05) (Figure 5D), while osteoclasts formed from the marrow cells of AC + LCn3PUFA rats were similar in size to those formed from bone marrow cells of control rats (Figure 5B,D). Furthermore, AC alone-treated rats were found to have significantly more nuclei within the osteoclasts when compared to osteoclasts formed from the bone marrow of control rats (Figure 5E). However, the changes observed in both osteoclast size and nuclei number in AC-treated rats were attenuated by LCn3PUFA supplementation (*p* < 0.05) (Figure 5D,E). These results suggest that, although LCn3PUFA supplementation had no effects on ex vivo osteoclast formation potential, it can reduce the size of osteoclasts formed and the number of nuclei within osteoclasts during AC treatment.

## 3. Discussion

Combination chemotherapy has been shown to be more effective in breast cancer treatment when compared to single agent used alone. However, women receiving combination treatment experienced more adverse effects. Chemotherapeutics such as taxane, adriamycin, 5-fluorouracil, cyclophosphamide, methotrexate, and cisplatin are known to cause an increase in bone resorption independent of bone metastasis and a deterioration in bone structure [17]. Adriamycin is a common anthracycline agent that is used widely in treating both early and late stage metastatic breast cancer. A clinical report indicated that premenopausal breast cancer patients treated with AC combination resulted in a lower bone mineral density and significant bone loss [18]. Animal studies have also reported that adriamycin exposure caused a reduction in bone formation due to reduced osteoblast differentiation [19], and can negatively affect bone trabecular microarchitecture and mechanical properties [20]. Using a rat AC treatment model mimicking the clinical setting, our recent animal study also revealed that 4 cycles of AC treatment were able to cause severe bone marrow damage and some reduction in trabecular bone volume [2]. Since anthracycline-based combination chemotherapy remains to be the backbone for treating breast cancer nowadays, it is vital to develop preventive strategies for alleviating bone marrow and bone toxicities that may affect quality of life post chemotherapy. Recently, several nutraceuticals (including LCn3PUFA) have been found to have some efficacies to promote bone health under physiological conditions or during methotrexate chemotherapy [21]. Consumption of LCn3PUFA has been suggested to attenuate aging-related bone loss and preventing bone loss in ovariectomized models [8,10]. In addition, daily LCn3PUFA supplementation at 375 mg/100 g body weight during short-term methotrexate chemotherapy was able to preserve bone formation, suppress bone resorption and marrow adiposity in young rats [11,12]. However, no previous studies have determined whether LCn3PUFA supplementation during anthracycline-based combination chemotherapy can maintain bone health in breast cancer survivors. Using a rat AC treatment model mimicking the clinical setting [2], the current study revealed that daily LCn3PUFA supplementation can protect against AC-induced bone marrow damage.

In the current study as well as in the previous study [2], rats were intravenously injected with the combination of 20 mg/kg cyclophosphamide and 2 mg/kg adriamycin once weekly for 4 weeks, dosages of which were similar to those converted from clinical doses in humans (cyclophosphamide 600 mg/m^2^ and adriamycin 60 mg/m^2^ for a total of 4 cycles). In addition, considering differences in drug metabolism rates between rats and humans, the dosages and the timing of dosing used in the current and the previous study [2] had also been adjusted basing on previous rat studies using these drugs [22,23], and basing on another AC model in female rats (used to study cardiotoxicity) in which the drugs were dosed once weekly for 4 weeks at similar but slightly higher doses (40 mg/kg cyclophosphamide + 3 mg/kg adriamycin) [24]. The current study revealed that rats were overall healthy in all treatment groups, despite a slight decrease in body weight gain after 3 cycles of AC treatment in AC alone group, which is consistent with the previous study [2]. Interestingly, starting from day 12, AC + LCn3PUFA-treated rats appeared to have a slightly higher weight gain when compared to AC-alone treated rats, suggesting potential benefits of LCn3PUFA in promoting overall health during AC chemotherapy.

Examination of the bone marrow environment in the current study revealed dramatic changes following 4 cycles of AC treatment. These changes include the depletion of bone marrow cells. Clinically, bone marrow suppression following cancer treatment is frequently reported, while some patients are known to have acute myelosuppression, others who receive chemotherapy and/or ionizing radiation may experience irreversible chronic bone marrow injuries [25]. A higher risk of bone marrow toxicity has also been reported in patients who received anthracycline-based regimens irrespective of the treatment cycles [1]. A significant limitation of the current study is that our data derived from ex vivo cell counting and histological examination have only indicated a decrease in bone marrow cell numbers or cellularity following AC treatment. Further studies will be required to investigate how AC chemotherapy impact on hematopoietic stem/progenitor cells and their clonogenic regeneration capacities.

Accompanying the myelosuppression discussed above is the increased marrow adiposity with significant infiltration of adipocytes, a phenomenon also commonly observed in cancer patients following irradiation or chemotherapy [26,27]. These findings are consistent with previous animal studies which reported treatments with methotrexate alone or CEF or AC combination were able to induce bone marrow toxicity in rats [2,3,28]. While mechanisms for the marrow injuries following chemotherapy are yet to be fully elucidated, one study has shown that the attenuated Wnt/β-catenin signaling in the bone may be mediating the enhanced bone marrow adipocyte accumulation following methotrexate treatment [29]. In addition, one study suggests that the phenomenon of bone marrow adiposity coupled with myelosuppression may be due to the imbalance between leptin, growth hormone/insulin-like growth factor and estrogen axis [30]. Further studies are required to examine whether these possibilities also apply in the AC chemotherapy setting.

Furthermore, it has also been suggested that bone marrow adipocytes do not just simply fill the marrow space but can act as negative regulators of the bone marrow microenvironment. Evidence has shown that adipocytes/stromal cells have direct functions on hematopoietic cells through their production of cytokines and adipokines, such as IL-6, TNF-α, and adiponectin which inhibit hematopoietic proliferation and activity, therefore negatively regulate bone marrow microenvironment [31,32]. In a mouse study, when arabinosylcytosine-C chemotherapy-induced adipogenesis was inhibited using an inhibitor, accelerated recovery of leukocyte counts, increased colony forming units and Ki67^+^Lin^−^Sca1^+^c-kit^+^ bone marrow cell population were observed [33], suggesting hematopoietic recovery is improved following arabinosylcytosine-C chemotherapy when adipogenesis was inhibited. However, the relationship between increased marrow adiposity and hematopoiesis still warrants further investigation following AC chemotherapy.

Our study also revealed that LCn3PUFA supplementation was able to partially prevent bone marrow damage from AC chemotherapy, as shown by the bone marrow cellularity recovery and reduced marrow adiposity. The benefits of LCn3PUFA in promoting hematopoiesis has also been reported in other animal studies. One study revealed that chronic intake of a LCn3PUFA-rich diet increases the abundance of hematopoietic stem cells in both bone marrow and spleen, in part via the activity of matrix metalloproteinase 12 [34]. Another study revealed that LCn3PUFA supplementation can restore rosiglitazone-induced bone marrow adiposity by suppressing pro-inflammatory cytokines and promoting anti-inflammatory cytokines [35]. Furthermore, LCn3PUFA supplementation can suppress methotrexate chemotherapy-induced bone marrow adiposity in young rats by suppressing adipocyte differentiation and expression of adipogenic genes PPARγ and FABP4 [12]. Overall, findings from this study and previous animal studies suggest daily LCn3PUFA supplementation may promote hematopoiesis while suppressing bone marrow adiposity that is known to negatively regulate bone marrow microenvironment under different circumstances. However, while the LCn3PUFA alone treatment showed a statistically non-significant trend of increasing bone marrow cellularity and decreasing adipocyte contents in control rats, and while the AC treatment had more substantial and statistically significant effects on bone marrow cellularity and in adiposity (opposite to effects of AC), we cannot rule out the possibility that the simple combination of these opposite effects (of AC and LCn3PUFA) may have contributed to the outcomes seen in the AC + LCn3PUFA group. Further studies are required to investigate the mechanisms for these treatment effects.

It is generally believed that the clinical observations of bone loss are primarily in post-menopausal women or in premenopausal women made amenorrheic by chemotherapy. It has been reported that women receiving anthracycline-based chemotherapy suffer from chemotherapy-induced amenorrhea and cause bone loss [36,37]. In postmenopausal patients who have progressive bone loss due to natural ovarian failure and aging, it has been also reported that adjuvant chemotherapy exacerbates estrogen deficiency, contributing to increased osteoporosis [38]. Furthermore, while accelerated bone loss can result from cancer treatment-induced endocrine defects [39], evidence has emerged and is emerging that breast cancer chemotherapy itself may also directly damage bone. A recent rat AC treatment model also in intact female rats has demonstrated that AC treatment can damage bone [2], and previous studies have shown that various cancer drugs including adriamycin, cyclophosphamide, 5-fluorouracil, and methotrexate individually or in combination (e.g., with CEF) can directly damage bone cells and interfere with bone formation [3,28,40,41,42,43]. Since cancer drugs can damage normal bone cells and bone regardless, and AC chemotherapy is given to both pre- and post-menopausal women, the current study has used an AC chemotherapy model in adult intact female rats. Furthermore, while further studies are required to examine whether AC chemotherapy can alter serum estrogen levels in treated rats, a recent rat CEF study showed that 6 cycles of CEF treatment only slightly and insignificantly reduced serum estrogen levels although the treated rats suffered from a significant loss of trabecular bone in tibia [3].

Clinical studies have reported that AC chemotherapy has negative impact on bone mineral density and can cause significant bone loss at several sites [18,44]. Our recent animal study also revealed significant trabecular bone loss with changes in trabeculae structures following 4 cycles of AC chemotherapy [2]. In the current study, no significant changes were observed in overall bone volume between all treatment groups. Interestingly, trabeculae structure examination revealed that the trabecular number was reduced following AC treatment, which was accompanied by increase in trabecular thickness and spacing, and a trend of statistically nonsignificant reduction in trabecular bone volume. These changes in trabecular structures were consistent with our previous findings [2].

Animal studies have reported that several cycles of combination chemotherapy involving the use of anthracyclines can negatively affect bone remodeling by either reducing numbers of bone surface lining cells (BLC) or promoting osteoclast formation or osteoclastic resorption [2,3]. The current study revealed no significant changes in densities of bone lining cells and osteoclasts following four cycles of AC treatment. However, despite the insignificant changes, the current study observed that LCn3PUFA supplementation appeared to have a trend to lower bone lining cell densities in both control and in AC-treated animals, the mechanism for which requires further investigation. In addition, a trend of osteoclast density reduction was observed in rats that received LCn3PUFA supplementation during AC treatment, suggesting daily LCn3PUFA intake may be able to suppress bone resorption during AC chemotherapy. The benefits of LCn3PUFA in reducing bone resorption in alleviating chemotherapy-induced bone damage has also been previously revealed both in clinical and animal studies [12,45]. It is suggested that LCn3PUFA can inhibit osteoclast formation through their anti-inflammatory properties [45], such as suppressing the mRNA expression of pro-osteoclastogenic and pro-inflammatory cytokines including RANKL receptor activator of nuclear factor kappa B (NF-κB) ligand], tumor necrosis factor-α (TNF-α), interleukin-6 (IL-1), and IL-6 [12].

In consistency with our histological findings, our ex vivo osteoclast formation assays revealed no significant changes in osteoclast formation potential of bone marrow cells across all treatment groups. However, the osteoclast size and the number of nuclei within osteoclasts were significantly increased in osteoclasts formed from bone marrow cells of AC-treated rats when compared to control, which was consistent with previous studies that reported bigger osteoclasts with more nuclei being formed from bone marrow cells of rats following several cycles of breast cancer AC or CEF chemotherapy [2,3]. Interestingly, osteoclasts formed ex vivo were found smaller in size and with fewer nuclei from the marrow cells of rats which received LCn3PUFA supplementation during AC treatment. It is known that osteoclast size and their nuclei numbers are positively associated with bone resorption potential and activity [46,47]. Therefore, our ex vivo data indirectly suggest that daily LCn3PUFA supplementation may attenuate osteoclast resorbing activity during breast cancer chemotherapy. In support of our ex vivo findings, one study reported that a LCn3PUFA diet was able to suppress the increased activity and number of osteoclasts in ovariectomized rats by suppressing activation of osteoclastogenesis transcription factor NF-κB, and subsequent downregulation of osteoclastogenic cytokines TNF-α, macrophage colony-stimulating factor (M-CSF), and RANKL [48]. Another animal study also revealed that daily LCn3PUFA supplementation can suppress methotrexate-induced expression of osteoclastogenesis-related cytokines and modulate inflammatory mediators, therefore suppressing bone resorption [12]. Although our results did not show a significant reduction in overall bone volume, it is possible that other breast cancer chemotherapy regimens may cause more severe damage to the bone by promoting more bone resorption, hence LCn3PUFA supplementation may still offer its therapeutic benefits in preventing bone loss during breast cancer chemotherapy.

The dose of LCn3PUFA given to the rats (3.75 g/kg/day, equivalent to consuming 0.5 mL/100 g/day) is high when compared to the recommended 3.4 g DHA + EPA/day for a 50–70 kg human, but is consistent with the typical efficacious dose for anti-inflammatory and cardiovascular benefits in numerous rodent studies. While further studies are needed to define the minimal effective dose of LCn3PUFA for protecting bone marrow and bone during AC chemotherapy for rats and humans, this dose was adopted from previous rat studies wherein LCn3PUFA supplementation during short-term methotrexate chemotherapy was able to preserve bone formation, suppress bone resorption and marrow adiposity in young rats [11,12], and emu oil at 1 mL/100 g/day was able to prevent 5-fluouracil chemotherapy-induced bone loss [49].

## 4. Materials and Methods 

### 4.1. Animal Trial

Female Sprague-Dawley rats of twelve-weeks old were randomly allocated into four groups (Table 1) receiving oral gavage of water or LCn3PUFA (ROPUFA^®^ 75-EE, an ethyl ester derivation from fish oil containing 42% EPA and 22% DHA) (DSM Nutritional Products, Kaiseraugst, Switzerland) at 0.5 mL/100 g/day (equivalent to consuming 375 mg of LCn3PUFA/100 g/day) [12]. After one week of pre-treatment, rats were intravenously injected with sterile water or the combination of AC once weekly for 4 weeks, comprising of 20 mg/kg cyclophosphamide (Baxter, Deerfield, IL, USA) and 2 mg/kg adriamycin (Ebewe, Unterach, Austria). Dosage chosen for cyclophosphamide and adriamycin were based on our previous study [2]. Daily oral gavage of water or LCn3PUFA continued throughout the trial and ended 1 day before specimen collection. By the end of fourth cycle, rats were euthanized by CO_2_ overdose and different specimens were collected for analysis. The animal procedures described above were approved by the Animal Ethics Committee of the SA Pathology (South Australia), Animal Ethics Approval Number: 129f 10 (approval date: 17 July 2013).

### 4.2. Specimen Collection

Both hind limbs were dissected and cleaned free of soft tissue. Left tibias were fixed in 10% formalin for 24 h, transferred to 70% ethanol for storage until ready for µ-CT analyses (see below). Tibias were wrapped in saline-soaked gauze prior to conducting µ-CT scanning. Following µ-CT, tibias were decalcified in Immunocal (Decal Corporation, Tallman, NY, USA) at 4 °C, processed and embedded in paraffin for 4 µm sectioning for histological analyses (see below). The right tibias and femurs and two front limbs were used to obtain bone marrow cells for isolation of bone marrow mononuclear cells (BMMNCs) using Lymphoprep [28]. BMMNCs were then plated and cultured overnight for the isolation of non-adherent haematopoietic cells for osteoclast formation assay (see below) [11].

### 4.3. Ex Vivo µ-CT

Trabecular bone volume and structures were assessed using the µ-CT scanner (Skyscan 1174, Kontich, Belgium). Briefly, trabecular bone region from 0–2 mm below the growth plate was selected for bone measurement analyses, which include trabecular bone volume/total volume ratio (BV/TV %), trabeculae thickness (mm), trabeculae number (per mm) and trabecular spacing (mm) [3].

### 4.4. Histological Analysis

Tibial paraffin sections (4 µm thick) were de-waxed and stained with H&E for analyses of bone lining cells, bone marrow adipocytes and bone marrow cellularity as described [42]. Sections were also stained with TRAP for the analyses of osteoclast density. To examine the effects of daily LCn3PUFA supplementation during AC chemotherapy on bone cells, densities of both bone lining cells (BLCs) and osteoclasts were measured along the trabeculae surface within the primary spongiosa. To examine the treatment effects on overall changes in the bone marrow, in the lower secondary spongiosa region (marrow rich area with areas containing haematopoietic cells), adipocytes were traced and scored as a percentage of total marrow area [50].

### 4.5. Ex Vivo Osteoclast Formation Assay

To assess the effects of daily LCn3PUFA supplementation during AC chemotherapy on the ability of osteoclast formation, an ex vivo osteoclast formation assay was performed. Briefly, bone marrow non-adherent cells were cultured overnight at 1.5 × 10^5^ cells/well in 96-well plate in basal medium supplemented with 10 ng/mL M-CSF (Peprotech, Rocky Hill, NJ, USA), followed by culturing in medium containing 10 ng/mL M-CSF and 30 ng/mL RANKL (Peprotech, Rocky Hill, NJ, USA). Culture was maintained for 8 days with M-CSF+RANKL medium changed every 3 days, and cells were fixed on day 9 for TRAP staining to identify osteoclasts. Apart from osteoclast density measurement, the average osteoclast size and number of nuclei per osteoclast were also determined as described [50].

### 4.6. Statistical Analysis

All results are presented as the means ± the standard error of the mean (SEM) and were analysed by non-parametric Kruskal-Wallis test. When significance (*p* < 0.05) was reached, the post hoc Dunn’s multiple comparison test was performed with GraphPad Prism 7 software (GraphPad Software Inc., Lo Jolla, CA, USA). In data graphs, * and ** above each bar indicate significance compared to the control group, and # and ## above horizontal bars reflect differences between connected groups. Significant values are indicated as: * or # = *p* < 0.05 and ** or ## = *p* < 0.01.

## 5. Conclusions

**Figure 6:**
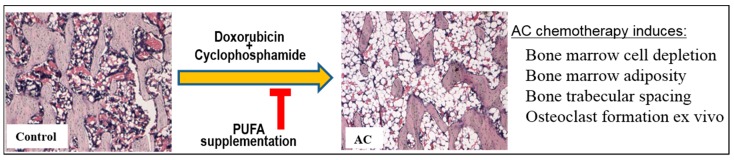
Long chain omega-3 polyunsaturated fatty acid (PUFA) supplementation helps to preserve bone and bone marrow microenvironment during adriamycin and cyclophosphamide (AC) combination chemotherapy. It protects against AC treatment-induced bone marrow cell depletion and adiposity and attenuates separation of bone trabeculae in rats and osteoclast formation ex vivo.

This study has examined the effects of dietary LCn3PUFA supplementation on long bones of rats subjected to 4 cycles of AC breast cancer chemotherapy. This study observed that 4 cycles of AC breast cancer chemotherapy increased trabecular bone separation. Although weekly AC treatment in this study did not cause a significant decrease in the overall bone volume or bone cell density, ex vivo results revealed increased osteoclast size and nuclear numbers in osteoclasts formed, indirectly suggesting an increased bone resorption potential following AC treatment. However, bone marrow was found more severely damaged following AC treatment, as indicated by depletion of bone marrow cells and an increase in marrow adiposity. Daily LCn3PUFA supplementation during AC chemotherapy maintained the bone marrow microenvironment by preserving bone marrow cellularity and suppressing marrow adiposity, and it was able to preserve the trabecular bone microstructure (Figure 6). Thus, LCn3PUFA supplementation showed promising effects in preventing AC chemotherapy-induced bone marrow damage, suggesting its therapeutic potential in alleviating marrow damage caused by breast cancer chemotherapy. We cannot exclude the possibilities that other breast chemotherapy regimens may induce more severe damage to both bone marrow and bone, hence further studies should be carried out with different breast cancer chemotherapy models. In addition, different doses of LCn3PUFA supplementation need to be examined to reveal the optimal dose required to relieve bone marrow and bone toxicities associated with breast cancer chemotherapy regimens.

## Figures and Tables

**Table 1:** List of treatment groups for the animal trial.

Groups	Injection	Gavage
Control (*n* = 6)	Sterile water	Sterile water
LCn3PUFA (*n* = 6)	Sterile water	LCn3PUFA
AC (*n* = 8)	AC	Sterile water
AC + LCn3PUFA (*n* = 8)	AC	LCn3PUFA

LCn3PUFA, omega-3 polyunsaturated fatty acids; AC, adriamycin + cyclophosphamide chemotherapy.

## References

[1] Nurgalieva Z., Liu C.C., Du X.L.L. (2011). Chemotherapy use and risk of bone marrow suppression in a large population-based cohort of older women with breast and ovarian cancer. Med. Oncol..

[2] Fan C.M., Georgiou K.R., Morris H.A., McKinnon R.A., Keefe D.M.K., Howe P.R., Xian C.J. (2017). Combination breast cancer chemotherapy with doxorubicin and cyclophosphamide damages bone and bone marrow in a female rat model. Breast Cancer Res. Treat..

[3] Fan C., Georgiou K.R., McKinnon R.A., Keefe D.M., Howe P.R., Xian C.J. (2016). Combination chemotherapy with cyclophosphamide, epirubicin and 5-fluorouracil causes trabecular bone loss, bone marrow cell depletion and marrow adiposity in female rats. J. Bone Miner. Metab..

[4] Anampa J., Makower D., Sparano J.A. (2015). Progress in adjuvant chemotherapy for breast cancer: An overview. BMC Med..

[5] Shapiro C.L., Manola J., Leboff M. (2001). Ovarian failure after adjuvant chemotherapy is associated with rapid bone loss in women with early-stage breast cancer. J. Clin. Oncol..

[6] Orchard T.S., Pan X., Cheek F., Ing S.W., Jackson R.D. (2012). A systematic review of omega-3 fatty acids and osteoporosis. Br. J. Nutr..

[7] Hogstrom M., Nordstrom P., Nordstrom A. (2007). n-3 Fatty acids are positively associated with peak bone mineral density and bone accrual in healthy men: The NO_2_ Study. Am. J. Clin. Nutr..

[8] Bullon P., Battino M., Varela-Lopez A., Perez-Lopez P., Granados-Principal S., Ramirez-Tortosa M.C., Ochoa J.J., Cordero M.D., Gonzalez-Alonso A., Ramirez-Tortosa C.L. (2013). Diets Based on Virgin Olive Oil or Fish Oil but Not on Sunflower Oil Prevent Age-Related Alveolar Bone Resorption by Mitochondrial-Related Mechanisms. PLoS ONE.

[9] Vanek C., Connor W.E. (2007). Do n-3 fatty acids prevent osteoporosis?. Am. J. Clin. Nutr..

[10] Matsushita H., Barrios J.A., Shea J.E., Miller S.C. (2008). Dietary fish oil results in a greater bone mass and bone formation indices in aged ovariectomized rats. J. Bone Miner. Metab..

[11] Raghu Nadhanan R.R., Skinner J., Chung R., Su Y.W., Howe P.R., Xian C.J. (2013). Supplementation with Fish Oil and Genistein, Individually or in Combination, Protects Bone against the Adverse Effects of Methotrexate Chemotherapy in Rats. PLoS ONE.

[12] Raghu Nadhanan R.R., Fan C.M., Su Y.W., Howe P.R.C., Xian C.J. (2014). Fish Oil in Comparison to Folinic Acid for Protection Against Adverse Effects of Methotrexate Chemotherapy on Bone. J. Orthop. Res..

[13] Almeida M., Laurent M.R., Dubois V., Claessens F., O’Brien C.A., Bouillon R., Vanderschueren D., Manolagas S.C. (2017). Estrogens and Androgens in Skeletal Physiology and Pathophysiology. Physiol. Rev..

[14] Fan C., Foster B.K., Wallace W.H., Xian C.J. (2011). Pathobiology and Prevention of Cancer Chemotherapy-Induced Bone Growth Arrest, Bone Loss, and Osteonecrosis. Curr. Mol. Med..

[15] Georgiou K.R., Hui S.K., Xian C.J. (2012). Regulatory pathways associated with bone loss and bone marrow adiposity caused by aging, chemotherapy, glucocorticoid therapy and radiotherapy. Am. J. Stem Cells.

[16] Mohamed A.M. (2008). An Overview of Bone Cells and Their Regulating Factors of Differentiation. Malays. J. Med. Sci..

[17] Kalder M., Hadji P. (2014). Breast Cancer and Osteoporosis-Management of Cancer Treatment-Induced Bone Loss in Postmenopausal Women with Breast Cancer. Breast Care.

[18] Hadji P., Ziller M., Maskow C., Albert U., Kalder M. (2009). The influence of chemotherapy on bone mineral density, quantitative ultrasonometry and bone turnover in pre-menopausal women with breast cancer. Eur. J. Cancer.

[19] Glackin C.A., Murray E.J., Murray S.S. (1992). Doxorubicin inhibits differentiation and enhances expression of the helix-loop-helix genes Id and mTwi in mouse osteoblastic cells. Biochem. Int..

[20] Fonseca H., Carvalho A., Esteves J., Esteves V.I., Moreira-Goncalves D., Duarte J. (2016). Effects of doxorubicin administration on bone strength and quality in sedentary and physically active Wistar rats. Osteoporos. Int..

[21] Su Y.W., Chen K.M., Hassanshahi M., Tang Q., Howe P.R., Xian C.J. (2017). Childhood cancer chemotherapy-induced bone damage: Pathobiology and protective effects of resveratrol and other nutraceuticals. Ann. N. Y. Acad. Sci..

[22] Moulin M., Piquereau J., Mateo P., Fortin D., Rucker-Martin C., Gressette M., Lefebvre F., Gresikova M., Solgadi A., Veksler V. (2015). Sexual dimorphism of doxorubicin-mediated cardiotoxicity: Potential role of energy metabolism remodeling. Circ. Heart Fail..

[23] Sato M., Shiozawa K., Uesugi T., Hiromatsu R., Fukuda M., Kitaura K., Minami T., Matsumoto S. (2009). Collaborative work on evaluation of ovarian toxicity. 7) Effects of 2- or 4-week repeated dose studies and fertility study of cyclophosphamide in female rats. J. Toxicol. Sci..

[24] Todorova V.K., Kaufmann Y., Klimberg V.S. (2011). Increased efficacy and reduced cardiotoxicity of metronomic treatment with cyclophosphamide in rat breast cancer. Anticancer Res..

[25] Wang Y., Probin V., Zhou D. (2006). Cancer therapy-induced residual bone marrow injury-Mechanisms of induction and implication for therapy. Curr. Cancer Ther. Rev..

[26] Ollivier L., Gerber S., Vanel D., Brisse H., Leclere J. (2006). Improving the interpretation of bone marrow imaging in cancer patients. Cancer Imaging.

[27] Shao L., Wang Y., Chang J., Luo Y., Meng A., Zhou D. (2013). Hematopoietic stem cell senescence and cancer therapy-induced long-term bone marrow injury. Transl. Cancer Res..

[28] Georgiou K.R., Scherer M.A., Fan C.M., Cool J.C., King T.J., Foster B.K., Xian C.J. (2012). Methotrexate chemotherapy reduces osteogenesis but increases adipogenic potential in the bone marrow. J. Cell. Physiol..

[29] Georgiou K.R., King T.J., Scherer M.A., Zhou H., Foster B.K., Xian C.J. (2012). Attenuated Wnt/b-catenin signalling mediates methotrexate chemotherapy-induced bone loss and marrow adiposity in rats. Bone.

[30] Błogowski W., Ratajczak M.Z., Zyżniewska-Banaszak E., Dołęgowska B., Starzyńska T. (2012). Adipose tissue as a potential source of hematopoietic stem/progenitor cells. Obesity.

[31] Naveiras O., Nardi V., Wenzel P.L., Hauschka P.V., Fahey F., Daley G.Q. (2009). Bone-marrow adipocytes as negative regulators of the haematopoietic microenvironment. Nature.

[32] Pronk C.J., Veiby O.P., Bryder D., Jacobsen S.E. (2011). Tumor necrosis factor restricts hematopoietic stem cell activity in mice: Involvement of two distinct receptors. J. Exp. Med..

[33] Zhu R.J., Wu M.Q., Li Z.J., Zhang Y., Liu K.Y. (2013). Hematopoietic recovery following chemotherapy is improved by BADGE-induced inhibition of adipogenesis. Int. J. Hematol..

[34] Xia S., Li X.P., Cheng L., Han M.T., Zhang M.M., Shao Q.X., Xu H.X., Qi L. (2015). Fish Oil-Rich Diet Promotes Hematopoiesis and Alters Hematopoietic Niche. Endocrinology.

[35] Rahman M.M., Halade G., Williams P. (2016). Omega-3 fatty acid-rich fish oil supplementation prevents rosiglitazone-induced osteopenia in insulin resistant C57BL/6 mice. FASEB J..

[36] Miller K.K., Klibanski A. (1999). Clinical review 106: Amenorrheic bone loss. J. Clin. Endocrinol. Metab..

[37] Berliere M., Dalenc F., Malingret N., Vindevogel A., Piette P., Roche H., Donnez J., Symann M., Kerger J., Machiels J.P. (2008). Incidence of reversible amenorrhea in women with breast cancer undergoing adjuvant anthracycline-based chemotherapy with or without docetaxel. BMC Cancer.

[38] Brufsky A. (2006). Management of cancer-treatment-induced bone loss in postmenopausal women undergoing adjuvant breast cancer therapy: A Z-FAST update. Semin. Oncol..

[39] Vehmanen L., Saarto T., Elomaa I., Makela P., Valimaki M., Blomqvist C. (2001). Long-term impact of chemotherapy-induced ovarian failure on bone mineral density (BMD) in premenopausal breast cancer patients. The effect of adjuvant clodronate treatment. Eur. J. Cancer.

[40] Fan C.M., Foster B.K., Hui S.K., Xian C.J. (2012). Prevention of bone growth defects, increased bone resorption and marrow adiposity with folinic acid in rats receiving long-term methotrexate. PLoS ONE.

[41] Xian C.J., Cool J.C., Paragius T., Foster B.K. (2006). Damage and recovery of the bone growth mechanism in young rats following 5-fluorouracil acute chemotherapy. J. Cell. Biochem..

[42] Xian C.J., Cool J.C., Scherer M.A., Macsai C.E., Fan C.M., Covino M., Foster B.K. (2007). Cellular mechanisms for methotrexate chemotherapy-induced bone growth defects. Bone.

[43] Xian C.J., Cool J.C., van Gangelen J., Foster B.K., Howarth G.S. (2007). Effects of Etoposide and cyclophosphamide acute chemotherapy on growth plate and metaphyseal bone in rats. Cancer Biol. Ther..

[44] Rana T., Chakrabarti A., Freeman M., Biswas S. (2013). Doxorubicin-Mediated Bone Loss in Breast Cancer Bone Metastases Is Driven by an Interplay between Oxidative Stress and Induction of TGF beta. PLoS ONE.

[45] Fabian C.J., Kimler B.F., Hursting S.D. (2015). Omega-3 fatty acids for breast cancer prevention and survivorship. Breast Cancer Res..

[46] Hu Y., Ek-Rylander B., Karlstrom E., Wendel M., Andersson G. (2008). Osteoclast size heterogeneity in rat long bones is associated with differences in adhesive ligand specificity. Exp. Cell Res..

[47] Fujita K., Iwasaki M., Ochi H., Fukuda T., Ma C.S., Miyamoto T., Takitani K., Negishi-Koga T., Sunamura S., Kodama T. (2012). Vitamin E decreases bone mass by stimulating osteoclast fusion. Nat. Med..

[48] Nakanishi A., Iitsuka N., Tsukamoto I. (2013). Fish oil suppresses bone resorption by inhibiting osteoclastogenesis through decreased expression of M-CSF, PU.1, MITF and RANK in ovariectomized rats. Mol. Med. Rep..

[49] Raghu Nadhanan R., Abimosleh S.M., Su Y.W., Scherer M.A., Howarth G.S., Xian C.J. (2012). Dietary emu oil supplementation suppresses 5-fluorouracil chemotherapy-induced inflammation, osteoclast formation, and bone loss. Am. J. Physiol. Endocrinol. Metab..

[50] Fan C.M., Cool J.C., Scherer M.A., Foster B.K., Shandala T., Tapp H., Xian C.J. (2009). Damaging effects of chronic low-dose methotrexate usage on primary bone formation in young rats and potential protective effects of folinic acid supplementary treatment. Bone.

